# Are performance indicators used for hospital quality management: a qualitative interview study amongst health professionals and quality managers in The Netherlands

**DOI:** 10.1186/s12913-016-1826-3

**Published:** 2016-10-13

**Authors:** Daan Botje, Guus ten Asbroek, Thomas Plochg, Helen Anema, Dionne S. Kringos, Claudia Fischer, Cordula Wagner, Niek S. Klazinga

**Affiliations:** 1 Amphia Hospital, Langendijk 75, P.O. box 90157, 4800 RA Breda, The Netherlands; 2 NIVEL, Netherlands Institute for Health Services Research, Utrecht, The Netherlands; 3 Ahti, Amsterdam Health & Technology Institute, Amsterdam, The Netherlands; 4 Department of Public Health, Academic Medical Center, University of Amsterdam, Amsterdam, The Netherlands; 5 Netherlands Public Health Federation, Utrecht, The Netherlands; 6 Philips Research, Cambridge, UK; 7 Department of Public and Occupational Health, EMGO Institute for Health and Care Research, VU University Medical Center, Amsterdam, The Netherlands

**Keywords:** Performance indicators, Quality management, Hospitals, Interview study

## Abstract

**Background:**

Hospitals are under increasing pressure to share indicator-based performance information. These indicators can also serve as a means to promote quality improvement and boost hospital performance. Our aim was to explore hospitals’ use of performance indicators for internal quality management activities.

**Methods:**

We conducted a qualitative interview study among 72 health professionals and quality managers in 14 acute care hospitals in The Netherlands. Concentrating on orthopaedic and oncology departments, our goal was to gain insight into data collection and use of performance indicators for two conditions: knee and hip replacement surgery and breast cancer surgery. The semi-structured interviews were recorded and summarised. Based on the data, themes were synthesised and the analyses were executed systematically by two analysts independently. The findings were validated through comparison.

**Results:**

The hospitals we investigated collect data for performance indicators in different ways. Similarly, these hospitals have different ways of using such data to support their quality management, while some do not seem to use the data for this purpose at all. Factors like ‘linking pin champions’, pro-active quality managers and engaged medical specialists seem to make a difference. In addition, a comprehensive hospital data infrastructure with electronic patient records and robust data collection software appears to be a prerequisite to produce reliable external performance indicators for internal quality improvement.

**Conclusions:**

Hospitals often fail to use performance indicators as a means to support internal quality management. Such data, then, are not used to its full potential. Hospitals are recommended to focus their human resource policy on ‘linking pin champions’, the engagement of professionals and a pro-active quality manager, and to invest in a comprehensive data infrastructure. Furthermore, the differences in data collection processes between Dutch hospitals make it difficult to draw comparisons between outcomes of performance indicators.

## Background

External accountability has become increasingly important over the last few years. As a result, hospitals are under increasing pressure to share indicator-based performance information with the government, regulatory bodies, health insurers and the general public. Hospital performance indicators facilitate patient choice and hospital-insurer contracts and promote public accountability. Public disclosure has already become common in, for example, the US and UK, where the data are increasingly based on clinical information from patient records [1]. Although patients do not seem to use publicly disclosed performance information to the full extent, it does appear to encourage hospitals to improve quality of care [2, 3].

There are major differences between countries in how the underlying data of performance indicators are collected and published. For example, in the Veterans Health Administration in the US, external contractors collect data from hospitals quarterly by auditing their electronic patient records [2]. In The Netherlands, on the other hand, indicators scores are self-reported by hospitals, which means that hospital employees collect and compute the data. The way performance indicators are computed affects their reliability and validity [4]. To our knowledge, the present study is the first qualitative interview study that actually targets the characteristics of performance indicator collection processes of various hospitals, in a so called ‘self-report country’. Given the increasing pressure of public accountability, there is an obvious need for empirical evidence regarding the methods of healthcare quality measurement.

Performance indicators for external accountability can also serve as a means to promote quality improvement and boost hospital performance [5]. For example, a natural experiment that covered thousands of hospitals in the US, pointed out that hospitals engaged in public reporting and pay-for-performance were more often involved in quality improvement projects [6]. External accountability, then, stimulates hospitals to put more effort in improving their performance.

Consequently, the assumption is that there should be a link between hospitals’ performance indicators for external accountability and the use of these indicators for internal quality management purposes. Hence, the processes of data collection, indicator calculation and reporting for external accountability must be linked to internal quality management processes. After all, monitoring specific indicators seems to improve the performance, while failing to do so does not [7]. For example, a European quantitative study shows that hospital CEOs feel compelled by pressures of external accountability to improve their quality management system [8].

It is to be assumed that, in order to facilitate effective data collection of performance indicators, responsibilities need to be assigned and procedures formalised. An important aspect of quality management is the availability of information about the processes of care delivery, as this provides input for improvement strategies. In a quantitative study in Dutch hospitals, however, the reliability and validity of the underlying data were found to be ambiguous due to the differences in data collection and data infrastructures [4], and the liberties taken in interpreting indicator definitions [9]. A quantitative study in The Netherlands shows that self-reported performance indicators for external accountability are largely implausible due to imprecise and inaccurate data collection [10]. A literature review shows that little is known about hospitals’ use of publicly released performance data for quality management [2]. In another literature review, De Vos and colleagues find that effective strategies for implementing performance indicators in quality improvement do seem to exist although that the internal use and effect of performance indicators varies [11]. Nevertheless, it remains unclear how hospitals produce performance indicator data and to what extent these indicators are used for internal quality management.

To that end, our aim was to explore hospitals’ use of performance indicators for internal quality management activities. In 14 hospitals in The Netherlands, we investigated the arrangements that were made for performance indicator data collection and to what extent they were used for internal quality management activities. The objectives were articulated in the following research questions:

1. What are the arrangements for data collection of performance indicators for external accountability in 14 hospitals?
2. To what extent are these indicators used for internal quality management activities?
3. Which factors explain possible differences between hospitals in how they use performance indicators for internal quality management?

## Methods

### Setting

In The Netherlands, performance indicators are submitted to various external parties. For example, the Dutch Health Care Inspectorate sets and monitors the minimum standards for quality, and requires hospitals to deliver approximately 62 performance indicators to allow patients to choose their preferred supplier. Additionally, every health insurer has their own set of performance indicators. For the Dutch Health Care Transparency Program, hospitals are obliged to publicly report approximately 115 performance indicators, covering 42 diseases. For each disease, a set of indicators was developed by expert groups on the basis of medical guidelines.

To get a better understanding of the data collection processes, we chose to focus on indicator sets of two conditions: 1) knee or hip replacement surgery, and 2) breast cancer surgery. These indicator sets were investigated at the orthopaedic and oncologic surgery departments, respectively. These conditions were selected because they have both department-specific as generic indicators such as nosocomial infections and blood transfusions. We focused on the indicators that were constructed within the hospital’s data infrastructure, excluding questionnaires on patient experiences. The corresponding data infrastructure and data collection processes for the indicators of these two conditions were considered to be generalizable for indicator sets of most conditions that are used for external accountability. Therefore, these two conditions were considered to be representative for the data collection and use in Dutch hospitals.

### Study sample and interviewees

This study was part of a larger study that aimed to investigate the validity, reliability and usability of performance indicators in Dutch hospitals [4, 10, 12]. All 97 hospitals in The Netherlands are private not-for-profit organisations. To better understand the practical use of performance indicators for quality management, we conducted interviews with key respondents such as quality managers and medical specialists. We chose interviews over a quantitative approach to learn more of the practical elements attributing positively or negatively to the use of performance indicators for quality management.

From the 42 participating hospitals in the larger study, a purposive sample of 22 hospitals was approached to participate in the current qualitative interview study, ensuring a balance between smaller and larger hospitals. Eventually 14 hospitals agreed to participate. These six teaching and eight non-teaching hospitals varied in size, geographical location and data infrastructure. In total, 11 departments of oncology and 11 departments of orthopaedic surgery agreed to participate. Heads of departments were asked to select senior professionals to participate voluntarily. A criterion was that the interviewees have a good overview of the arrangements and the use of performance indicators for internal quality management. We interviewed quality managers in each participating hospital to get a better understanding of the quality management system at hospital level. To learn more of day-to-day practice at departmental level, we aimed to talk to medical specialists, nurses and quality management staff at each participating department. Eventually, we were able to conduct 72 semi-structured interviews with 21 medical specialists (11 orthopaedic and 10 oncologic surgeons), 13 nurses, 31 employees of quality management staff (quality managers, data managers) and seven other employees (manager, department heads) between January and June 2012 (Table 1).

**Table 1:** Overview of 72 interviewees

Hospital	Affiliation	Quality management staff	Medical specialist	Nurse	Other
1	Hospital level	3			
	Orthopaedic		1		
	Oncology		1		
2	Hospital level	1			
3	Hospital level	2			
	Orthopaedic		1		1
	Oncology		1	1	1
4	Hospital level	2			
	Orthopaedic		1		
	Oncology		1		
5	Hospital level	2			
	Orthopaedic		1	1	
	Oncology		1	1	
6	Hospital level	2			
	Orthopaedic		1		
	Oncology		1		
7	Hospital level	2			
	Orthopaedic		1		
	Oncology			1	
8	Hospital level	1			
	Oncology		1		
9	Hospital level	3			
	Orthopaedic		1	1	
10	Hospital level	2			
	Orthopaedic	1	1	1	1
	Oncology	1	1	1	
11	Hospital level	2			
	Orthopaedic		1	1	
12	Hospital level	3			1
	Orthopaedic		1		1
	Oncology		1	2	
13	Hospital level	2			1
	Orthopaedic		1	1	
	Oncology		1	1	
14	Hospital level	2			1
	Oncology		1	1	
	Total	31	21	13	7

### Content and conduct of interviews

In the semi-structured interviews we used an interview guide (see Appendix) that aimed to learn more about the data collection processes, indicator score calculation methods and the influencing factors. Quality managers were asked about how the performance indicators were used at hospital level, whilst professionals were asked about the use at department level. The interviews took 30 to 60 min and were conducted at the respective hospitals. DB, GtA and HA are health services researchers and GtA also has a background in nursing. For the purpose of open discussions, interviewees were assured confidentiality.

### Coding and analysis

Our study design was neither inductive (developing theory) nor deductive (testing theory). Our aim was primarily to explore the practice-variation of quality management and to understand the underlying mechanisms that lead to this variation. To get a sense of the practice variation, we chose to summarize instead of transcribe the audiotaped interviews. We considered that the summaries suffice in exploring the practice-variation and to better understand the influencing factors attributing positively or negatively to the use of performance indicators for quality management. A previous study showed that audiotapes and field notes allow researchers to determine if the summary is an accurate reflection of the interaction in the interview [13]. To assure the accuracy of our summaries, DB, GtA and HA each summarised, compared and discussed five interviews. After consensus was reached about the summaries, DB summarised the other interviews. The interviews were analysed using predetermined categories: 1) tasks and responsibilities about data collection processes are appointed to stakeholders, 2) procedures are formalised in order to determine who is doing what at a given time, and 3) to what extent performance indicators for external accountability were used for internal quality management. We considered these categories to reflect structure (tasks and responsibilities), process (formalised procedures) and outcome (actual use in practice) aspects of a mature quality management system. We defined a number of elements that we could attribute to each category. For each element we formulated a code. DB coded all summaries using MAXQDA 7 software. Subsequently, DB and GtA independently analysed the interviews to determine which of the elements of each category was mentioned during the interviews. The combination of these elements allowed us to learn more about how performance indicator data were collected and used for quality management in each hospital. To determine practice-variation between hospitals and departments, DB and GtA gave positive or negative scores when the abovementioned responsibilities and procedures were in place or not. DB and GtA discussed their scores till consensus was reached. In the results section, findings are illustrated by selected quotes that are translated into English.

## Results

Our key finding was that hospitals had different ways of arranging performance indicator data collection, and using -and not using- it for internal quality management. The level of formalisation of responsibilities and data collection processes were not in tune with the use for internal quality management activities.

### Arrangements for data collection of performance indicators for external accountability

Formal arrangements were made for the tasks and responsibilities in the data collection processes. Medical specialists and nurses were responsible for the registration in patient charts, although some indicated it is a burden to register all required data elements. They felt that every minute spent on administration is a minute spent less on patients. As a result, some medical specialists chose to spend their time predominantly on patients, leading to less complete patient records. Only a few hospitals made arrangements concerning registration completeness.

“For each indicator we appointed someone who is responsible for it, together with their supporting staff.” (quality manager, H3)

The formalisation of procedures was generally achieved by setting up protocols. In these protocols, tasks for data collection are specified, responsibilities are appointed to individuals and the processes are reviewed regularly. For example, one hospital formalised these tasks and responsibilities by adding the names of the responsible persons to certain tasks. Subsequently, when a task appeared to need more attention, the person responsible was easily identified and reminded of that task.

In other hospitals, however, the data collection processes were not formalised. This meant that the quality manager would not have an existing data set at the annual indictor scores submission for external accountability. Quality manager were therefore preoccupied to retrieve the data from patient charts and to calculate the indicator scores in the preceding months. In general, data came from different sources, which make it difficult to collect these data for calculating indicator scores. Subsequently, there were hospitals that decided to report 100 % compliance on several indicators without calculating the actual score.

“At patient level you can assume that a treatment is given when there is a protocol for it. So a 100 % score for that indicator is never checked.” (quality management staff, H10)

“For some of the indicators I report 100 %, because we have protocols for them.” (quality manager, H12)

“Antibiotics is always given prior to incision according to protocol. I’m absolutely certain about that! You cannot continue to the next shackle in the chain without checking if everything is okay. However, I have no idea what our actual performance is on this indicator.” (orthopaedic surgeon, H13)

### Using performance indicators for internal quality management

#### Use for quality management at departmental level (oncology and orthopaedic surgery)

At an oncology department, performance indicator data are used twice a year in a meeting with all employees who were involved in the care for breast cancer patients, and the medical specialists have six meetings a year involving quality performance. When their performance appeared to stay behind, they tried to improve the underlying processes.

“About two years ago we reported an estimated score of 100 % for antibiotics before surgery. When we actually started measuring, it appeared to be 50 %. Since then we improved our procedures and now there is around 90 % compliance.” (quality manager, H11)

However, indicators are not always used for quality management.

“I do not think the collected data play an important role in my work, because I usually know more or less how well things are going; this is how we do things around here.” (oncology surgeon, H14)

A similar perception was observed in other hospitals. In one of the teaching hospitals, the orthopaedic surgeon indicated that one of their research assistants collects data for research projects, but that the findings are not shared with the department before the results are published in a scientific journal. The department does not have performance indicator data other than the research data.

“Our quality manager collects the data once a year. In the meantime, we do not know if we are performing well according to the guidelines. Once we receive the information from the quality manager, and it appears that we perform below par, then nothing changes.” (orthopaedic surgeon, H1)

#### Use for quality management at hospital level

It was mentioned that indicators were used for quality management at hospital level. Generally, the quality manager draws up a report to get the official approval of the executive board to submit it annually for external accountability. A quality manager indicated that the external accountability lead to a change in the mind set of employees.

“After a low score on a national hospital ranking, our doctors and nurses became more aware of the importance of performance indicators, and they became more cooperative in terms of data registration and collection.” (quality manager, H4)

Other executive boards also used indicators for internal quality management. For example, performance indicators were discussed every three months in a meeting with the department heads.

### Factors explaining the differences between hospitals in using performance indicators for internal quality management

#### Champions as linking pins

In a few cases, employees considered it their duty to collect data and to share it with their colleagues. These ‘champions’ can be considered the linking pins between data collection and its use for quality management activities, i.e. ‘linking pin champions’. One of the interviewed nurses was such a linking pin champion. She was very dedicated to collect the data correctly, so she spent much time after working hours to manually copying specific data from the patient charts into a self-made Excel sheet. Then, she used this Excel sheet to inform the medical specialists at her department about their performance. In the interview, however, she acknowledged that this was not a sustainable situation because it relied solely on her involvement.

“When I would get promoted to another function or transferred to another department, then there is no one who knows where to find the data and no data will be given to the medical specialists.” (nurse at oncology department, H7)

In another hospital, a nurse with an IT background aligned the nurses’ electronic patient record when guidelines were updated. For example, when an extra step was added to a guideline, the nurse added this step into the electronic patient record. And because it was an important step, the nurse made it obligatory to fill it in so that the nurses could not forget to work according the new guideline.

“The nurses are now working with electronic patient records, but the orthopaedic surgeons are still working with paper records. But that is something that I’m working on to change.” (orthopaedic nurse, H11)

#### A pro-active role of the quality manager

The interviews showed that quality managers carried out their role differently. Some of them merely collected data for the annual reporting of performance indicators for external accountability, while others were more pro-active. There was a quality manager who had a reactive approach.

“On the oncology department the improvements are initiated by some of the employees themselves. My role is just to collect the data.” (quality manager, H14)

Another quality manager was more pro-active and pushed the executive board’s quality agenda.

“I think it’s important that the board knows about our quality performance. Therefore I frequently make an update, print it out and put it on the CEO’s desk.” (quality manager, H13)

The role and position of the quality manager determined their influence on professionals to be held accountable for their quality performance, while influencing the executive board’s quality agenda on the same time. In other words, quality managers either merely *pulled* data from the patient records for external accountability, or also *pushed* the quality management agenda. In hospitals where the quality manager was more pro-active, data appeared to be used more systematically for quality management activities, even when data collection arrangements were poor.

#### Engagement of medical specialists

The use of indicators for quality management at departmental level seemed to largely depend on the engagement of medical specialists.

“Medical specialists in our hospital really feel that they are responsible for the outcomes of the indicators.” (quality manager, H5)

“Registration in patient charts is part of care delivery.” (oncology surgeon, H8)

However, some medical specialists were sceptical about the validity of few indicators. In practice, they only used indicators that were perceived interestingly. For example, one orthopaedic surgeon indicated that the timing of administering antibiotics prior to hip or knee replacement surgery was not relevant:

“This is not a good indicator. When we score poorly on it, then I do not change anything because I do not think this indicator is important. Indicators should focus on results, such as the functionality of the patient one year after surgery.” (orthopaedic surgeon, H1)

“If they [fellow orthopaedic surgeons] do not see the link between indicators and the ‘real’ quality of care, then it is hard to convince them to register the underlying data properly.” (orthopaedic surgeon, H4)

#### Diversity in data infrastructures

Hospitals are free to develop their own data infrastructure. We observed 14 different types of data infrastructures in 14 different hospitals. Patient records were either paper-based, electronic or a combination of both. Even where patient records were completely electronic, the type of software often differed between departments. Hospitals with a cohesive and homogenous electronic data infrastructure, performance indicator scores could be calculated ‘with a click of a button’. A less robust data infrastructure, however, has consequences for the time and efforts to collect performance indicator data correctly.

“We investigated how to improve the communication between different data systems, and it will cost hundreds of thousands of euros to get it done.” (quality management staff, H2)

“We do not have electronic patient records, so it is difficult to collect data from all the different sources.” (quality management staff, H6)

“In this hospital we have one electronic patient record system. To collect the data we have to write the command in our software and then it is just a matter of ‘a click of a button’.” (quality management staff, H9).

Additionally, the more manual labour is needed to collect the data from different sources, the more chances there are in general for making mistakes.

## Discussion

### Summary of main findings

In this qualitative study our aim was to gain more insight into the arrangements of data collection of performance indicators for external accountability and its use for internal quality management in 14 hospitals in The Netherlands. Our findings show that hospitals have different ways of collecting data for performance indicators and different ways of using- and not using- these data for internal quality management. Factors such as ‘linking pin champions’, pro-active quality managers and engaged medical specialists seem to make a difference. In addition, a homogenous data infrastructure appears to facilitate the production of usable performance indicators.

### Diversity in data collection

The investigated hospitals use different data collection processes. Some hospitals have made arrangements for the collection of performance indicator data; others rely on individuals who are tasked with collecting the corresponding data. Additionally, some hospitals determine the indicator scores by estimation (reporting 100 % because of protocol), instead of calculating the scores based on specific numerator and denominator. Reporting a 100 % compliance score that is not based on data, does not reflect the quality of care that was actually achieved. As a result, it becomes very difficult to interpret hospital performance, possibly reducing the vaunted effect of market competition. Allowing hospitals to report these kinds of estimated compliance scores hinders the primary goal of transparency: to improve the quality of care. In the light of the usefulness of performance indicators for *external accountability*, Van Dishoeck and colleagues stipulate that outcome indicators –and rankings especially- are not suitable for hospital comparison because the within-hospital variance appears to be greater than the between-hospitals variance [14]. Additionally, the validity of the indicators can be ambiguous due to different interpretations of definitions [10]. For example, the Health Care Inspectorate and the Dutch Health Care Transparency Program employ different definitions of the indicator ‘tumour positive surgical margin following breast-conserving surgery’, having different notions of how much tumour tissue is acceptable in the surgical margin [15].

The fact that the data for external accountability are self-reported can lead to hospitals getting caught in a transparency paradox: the more is measured, the more can be discussed. Moreover, the diversity of performance indicators also has implications for patients, making it difficult for them to make well-informed choices between hospitals when the indicators do not reliably reflect the hospitals’ actual performances. Performance indicators should be acceptable, feasible, reliable, valid and sensitive to change [16]. In order for performance indicators to be comparable between hospitals and a useful tool for patients, the underlying data infrastructure and the arrangements for data collection need to be formalised.

### Linking internal quality management to external accountability

The results of our study show that the potential of performance indicators to support internal quality management activities remains untapped. This runs contrary to the expectation that hospitals’ obligation to report quality performance at least once a year would lead to an increased use of data for internal quality management. After all, monitoring the performance of health care organisations should be an integral part of modern health care [17].

The hospitals in our study have different arrangements and different levels of formalisation with regards to their data collection processes. Even though some hospitals have such processes in place, the usability of the indicators appears to rely on medical specialists’ appreciation of their value. When medical specialists consider the indicator data to be useless, they tend to spend less time and effort on registration in patient records, resulting in poor data quality. A patient record review study in Dutch hospitals shows that the quality of the data registered in patient records was associated with the quality of care delivered to patients [18].

To break away from this vicious circle, the added value of performance indicators should be made clear to medical specialists, provided that only the most relevant indicators are selected. There has been an extensive international debate about which indicators represent quality of care and which combination of indicators validly represents hospital-wide performance with regards to quality of care [19]. Once the added value of data widely recognised, there will be an incentive to optimise the quality of the data. This will result in reliable performance indicators that can be used for quality management.

In cases where data collection processes are not embedded in formalised protocols, factors such as ‘linking pin champions’ may still make it possible to produce indicator data and to use it as input for quality management. Individuals are different in their ability to champion change, which makes it crucial for organizations to identify those front runners who are likely to react positively to innovations [20]. Additionally, professionals are more likely to adopt innovations effectively in the presence of champions as boundary spanners [21]. Apparently, linking pin champions can make a difference as front runners and boundary spanners to adopt new practices, even when processes are not formalised. Moreover, the engagement of medical specialists played a pivotal role in the use of performance indicators at the hospitals we investigated. They appeared to select the indicators they considered to be useful for internal quality management, and based that selection on perceived validity and reliability. Previous research already suggested that quality measures should be meaningful, and that such measures should be part of the clinical workflow [22].

Another trend in the Dutch healthcare system is the vast increase of the amount of performance indicators that hospitals are required to produce. While the Health Care Inspectorate has their own set of indicators, all the insurance companies, patient organisations and condition-specific national bodies are trying to play their part in the transparency dogma by requiring information about an increasing number of performance indicators. These additional indicators are often only slightly different from the existing indicators. This phenomenon feeds the discussion about the disproportionate amount of time doctors have to spend on administrative tasks. This calls for more efficiency, and thus to make better use of the existing indicators, both externally and internally. In Denmark, for example, there is a centralised database with which all Danish hospitals are obliged to share performance indicator data. Subsequently, these data are used to inform the public and to give feedback to the hospitals about their performances. This nation-wide indicator program seems to improve the quality of care [23].

### Strengths and limitations

To maximise representativeness, we incorporated a purposive sample of 14 hospitals. Following the 72 semi-structured interviews, we were able to develop a rich qualitative data set. We chose to summarise the interviews instead of transcribing them. As a result, some finer details about the exact processes may have become lost; however, considering the explorative approach of our study, the summaries did allow us to get a general idea about the practical use of indicators in quality management. We focused on the data collection process and use of a set of performance indicators for breast cancer and knee and hip replacements. It is possible that performance indicators for other conditions are collected and used slightly differently. Due to the underlying care processes of these conditions, the corresponding data infrastructure and the inclusion of generic indicators of other hospital-wide health care processes such as nosocomial infections and blood transfusions, findings for these two conditions were considered to be generalizable for other conditions and their concomitant indicators. Therefore, these indicator sets were useful in better understanding the data collection and use for quality management in Dutch hospitals.

## Conclusions

In The Netherlands, hospitals are obliged to report performance indicators for external accountability at least once a year. In our qualitative interview study, we found that performance indicators for external accountability are underused in internal quality management. For some hospitals, it seems, window dressing is more important than actual performance. Pressure caused by external accountability does not automatically result in increased use of indicators for internal quality management activities. In order to use performance indicators for internal quality management, hospitals are recommended to focus their human resource policy on ‘linking pin champions’, the engagement of professionals and a pro-active quality manager. Executive boards can give support by implementing a homogenous data infrastructure that allows performance indicators to be collected and calculated based on reliable data. Future research should focus on the sustainable implementation of performance indicator data in quality management activities. The diverse practices of data collection and reporting could lead to major comparability problems between hospitals. Delivering useful performance indicator data for external accountability takes a great deal of time and effort. It would be a waste not to use it to support internal quality management in its effort to improve the quality of care.

## Appendix

The appendix contains the interview guide that was used during the semi-structured qualitative interviews

**Table 2:** Interview guide

Topic	Example of questions
Quality policy	Are indicators part of the quarterly meetings with the executive board?
	Are the indicators discussed similar to the indicators used for external accountability?
	How are indicators fed back to the speciality groups or the heads of departments?
	How are indicators used for improvement projects?
Data registration	How are patient data registered (electronically/on paper/etc.)?
	Can you give a description of the protocol on data registration?
	What can you say about the quality of data registration, in terms of completeness/accuracy/timeliness/mistakes?
	Who checks these registered data, or reports these data, and to whom?
Data collection	How do you apply the definitions of indicators, such as *irradicality* or the completion time of e.g. door-to-needle?
	How are data collected?
	Who checks these collected data, or reports these data, and to whom?
Feedback	How are you informed about the performance of your specialty group/department?
	Which information is used for feedback?
Use	What actions follow from this feedback?
	How are these actions evaluated?
